# Anterior fusion surgery with overcorrection in the treatment of adolescent idiopathic scoliosis with Lenke 1 AR curve type: how to achieve overcorrection and its effect on postoperative spinal alignment

**DOI:** 10.1186/s12891-023-06989-4

**Published:** 2023-11-07

**Authors:** Nobuki Tanaka, Satoshi Inami, Hiroshi Moridaira, Tsuyoshi Sorimachi, Haruki Ueda, Hiromichi Aoki, Satoshi Takada, Yutaka Nohara, Hirotaka Haro, Hiroshi Taneichi

**Affiliations:** 1 Department of Orthopaedic Surgery, Dokkyo Medical University, 880 Kitakobayashi, Mibu-Machi, Shimotuga-Gun, Tochigi, 321-0293 Japan; 2 Department of Orthopaedic Surgery, University of Yamanashi, Chuo, Japan; 3 Department of Orthopaedic Surgery, Gotenyama Hospital, Kanuma, Japan

**Keywords:** Adolescent idiopathic scoliosis, Overcorrection, Lenke classification, 1AR, Anterior surgery

## Abstract

**Background:**

The efficacy of anterior fusion with overcorrection in the instrumented vertebra for Lenke 1 AR type curves has been reported, but how to achieve overcorrection and how overcorrection affects spinal alignment are unclear. The purpose of this study was to identify the factors that cause overcorrection, and to investigate how overcorrection affects postoperative spinal alignment in the surgical treatment of Lenke 1 AR type curves.

**Methods:**

Patients who had anterior surgery for a Lenke type 1 or 2 and lumbar modifier AR (L4 vertebral tilt to the right) type scoliosis and minimum 2-year follow-up were included. The radiographic data were measured at preoperative, postoperative 1 month, and final follow-up. The UIV-LIV Cobb angle was determined as the Cobb angle between the upper instrumented vertebra (UIV) and the lower instrumented vertebra (LIV), and a negative number for this angle was considered overcorrection. The screw angle was determined to be the sum of the angle formed by the screw axis and the lower and upper endplates in the LIV and UIV, respectively. The change (Δ) in the parameters from postoperative to final follow-up was calculated. The relationships between the UIV-LIV Cobb angle and other radiographic parameters were evaluated by linear regression analyses.

**Results:**

Fourteen patients met the inclusion criteria. Their median age was 15.5 years, and the median follow-up period was 53.6 months. The median UIV-LIV Cobb angle was –1.4° at postoperative 1 month. The median screw angle was 4.7°, and overcorrection was achieved in 11 (79%) cases at postoperative 1 month. The screw angle (*r*^*2*^ = 0.42, *p* = 0.012) and Δ FDUV-CSVL (the deviation of the first distal uninstrumented vertebra from the central sacral vertical line, *r*^*2*^ = 0.53, *p* = 0.003) were significantly correlated with the UIV-LIV Cobb angle.

**Conclusions:**

Screw placement in the UIV and LIV not parallel to the endplate, but angled, was an effective method to facilitate overcorrection in the instrumented vertebrae. The results of the present study suggest that overcorrection could bring spontaneous improvement of coronal balance below the instrumented segment during the postoperative period.

## Background

The Lenke classification is widely used to define curve types in adolescent idiopathic scoliosis (AIS) [1, 2], and it is reported that Lenke 1A type has subtypes depending on the direction of L4 vertebral tilt, i.e. 1AL to the left and 1AR to the right [3]. Adding-on is one of the postoperative complications of surgical treatment for Lenke 1A type scoliosis, which is caused by more proximal lower instrumented vertebra (LIV) selection than the ideal LIV position [4, 5]. Some studies noted that 1AR curves require a more distal fusion than 1AL curves [3, 6], and Cho et al. reported that 1AR curves were 2.2 times more likely to experience adding-on than 1AL curves, and they recommended fusing distally to 1 to 2 levels above the stable vertebra [6].

On the other hand, Inami et al. reported that anterior surgery for the 1AR curve could minimize the distal extent of the instrumented fusion without adding-on [7]. Their median LIV level of anterior surgery of 3 levels above the stable vertebra should cause adding-on according to Cho’s report [6], but no adding-on was present. They suggested that the reason for the good surgical result was that short fusion and overcorrection between the upper instrumented vertebra (UIV) and the LIV would maintain trunk balance. Some studies of anterior spinal fusion in thoracolumbar/lumbar idiopathic scoliosis stated that the advantage of anterior fusion was shorter fusion at a caudal fusion level than posterior fusion [8–10]. Bernstein et al. reported good surgical results of short anterior fusion with overcorrection in the instrumented vertebra for thoracolumbar scoliosis [8]. In the two studies by Inami [7] and Bernstein [8], the overcorrection in the anterior instrumented vertebra was considered the key factor for successful short anterior fusion, but how to achieve overcorrection and how overcorrection affects spinal alignment in the postoperative period were unclear.

The purpose of this study was to identify the factors related to overcorrection in the instrumented vertebra, and to investigate how overcorrection affects postoperative spinal alignment in the surgical treatment for a Lenke type 1 AR type curve.

## Methods

This study was a single-institution, retrospective analysis of patients undergoing anterior surgery for AIS with a Lenke type 1 or 2, and lumbar modifier AR type, i.e., the L4 vertebra was determined to be tilted to the right as seen on a posteroanterior standing radiograph [3]. Patients whose surgery was performed between 2008 and 2017 and had minimum 2-year follow up were included. The present study was conducted with institutional review board approval. Anterior surgery was chosen in cases with the apex vertebra of the main thoracic curve at or below the T10/11 disc, and the anterior and posterior combined approach was chosen in Lenke type 2 curves with the apex vertebra of the main thoracic curve at or below the T10/11 disc. In the combined approach, the main thoracic curve was first corrected by anterior surgery, and then posterior surgery was performed. The role of posterior surgery was to correct the only proximal thoracic curve and not the main thoracic curve. Patients with the apex vertebra of the main thoracic curve above T10/11 disc were treated by posterior surgery and were therefore not included in this study.

### Anterior surgery procedure

**Fig. 1:**
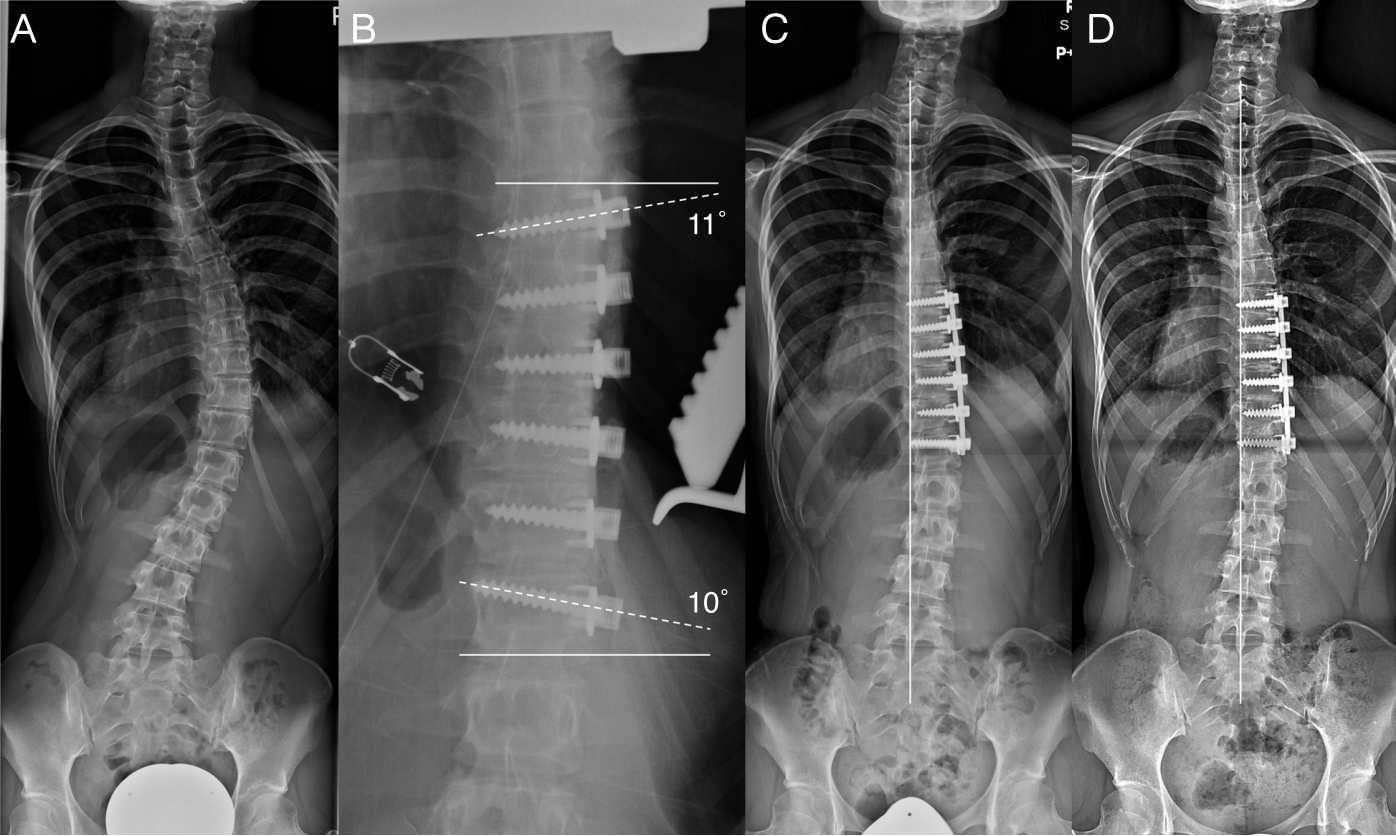
Case example of a 16-year-old female patient with Lenke 1 AR type curve. **A** before surgery, Cobb angle of the main thoracic curve is 47°. **B** intraoperative radiograph, the screw angle is 21° (i.e. angle formed by the screw axis and the upper endplate in the UIV is 11°, and the angle formed by the screw axis and the lower endplate in the LIV is 10°. The line represents the slope of endplate, and the dotted line represents the screw axis.). **C** at 1 month after surgery, anterior surgery has achieved overcorrection with the UIV-LIV Cobb angle of -11°, and FDUV-CSVL is 18 mm. The line is the central sacral vertical line. **D** at 4 years after surgery, FDUV-CSVL has gradually decreased over the postoperative period to 13 mm. The line is the central sacral vertical line

Surgical instrumentation included vertebral plates, monoaxial screws, and 4.5-mm titanium rods. We prefer to avoid proximal exposure as far as invasion to the muscles around the scapula, so the UIV was set at or below T8. Additionally, in order to leave more mobile intervertebral discs, the LIV was set at or above L2. The rod was not bent and was set into the screw head with cantilever force from the LIV to the UIV. The monoaxial screws in the UIV and LIV were placed not parallel to the endplate, but angled to facilitate overcorrection between the UIV and LIV. The proximal screw was descending in the UIV, and the distal screw was ascending in the LIV. It was expected that angled placement of screws makes overcorrection possible, because by securing a monoaxial screw rectangular to the rod, the LIV and UIV would tilt more to the direction of overcorrection (Fig. 1B, C).

### Evaluation

Radiographic parameters including main thoracic Cobb angles, thoracic apical vertebral translation, C7-central sacral vertical line (CSVL), which was the deviation of the C7 vertebra from the CSVL, L4 tilt, which was the angle formed by its superior endplate and a horizontal line, disc angulation above the UIV, disc angulation below the LIV, and first distal uninstrumented vertebra (FDUV)-CSVL, which was the deviation of the FDUV from the CSVL, were measured at preoperative, postoperative 1 month, and final follow-up. Sagittal parameters including T5-12 thoracic kyphosis (TK), T10-L2 thoracolumbar kyphosis (TLK), T12-S1 lumbar lordosis (LL), were also measured at preoperative and final follow-up. These radiographic data consisted of full-length standing coronal and sagittal radiographs obtained with a long 36-in cassette and a 72-in distance with the radiograph tube. The flexibility of the main thoracic curve was evaluated on side bending preoperatively. The UIV-LIV Cobb angle, which was the Cobb angle between the UIV and the LIV, was measured at postoperative 1 month and at final follow-up. The changes (Δs) in the coronal parameters from postoperative 1 month to final follow-up were also calculated. Screw angle was determined to be the sum of the angle formed by the screw axis and the endplate (i.e. upper endplate in the UIV, lower endplate in the LIV) were measured on intraoperative radiographs taken just after screw placement (Fig. 1B). A negative number indicated either a tilt or translation or angle open to the left in the coronal plane. Screw angle was an exception in that a positive number indicated an angle open to the left. Overcorrection was determined to be a negative number for the UIV-LIV Cobb angle. Adding-on was defined by the criterion of Wang et al. [4] at final follow-up. All radiographic measurements were conducted using measurement software (Centricity™ Enterprise Web, version 3.0, GE Healthcare Japan, Tokyo, Japan).

### Statistical analysis

Medians and ranges were calculated for continuous variables. The screw angle was compared between an overcorrection group (i.e. participants with overcorrection at postoperative 1 month) and a non-overcorrection group by Wilcoxon single-rank test. To identify the factors related to overcorrection, the UIV-LIV Cobb angle and preoperative thoracic curve-related parameters (i.e. Cobb angle, flexibility, apical translation) and intraoperative parameters (i.e. screw angle) were evaluated by simple linear regression analyses. Furthermore, to evaluate the effect of overcorrection, radiographic parameters at postoperative 1 month and final follow-up and Δ values were evaluated by simple linear regression analyses. Statistical analyses were performed using the JMP software package (JMP 14.2, SAS, Cary, NC), and the level of significance was set at 0.05.

## Results

### Patient data

Fourteen patients (11 females, 11 Lenke type 1, 3 Lenke type 2) met the inclusion criteria. The anterior approach was performed in 11 cases and the combined approach was performed in 3 cases. The median (range) age was 15.5 (12–18) years, and the median (range) follow-up period was 53.6 (24–108) months. The distribution of the apex levels was T10/11 in 3 patients, T11 in 8, and T11/12 in 3. The UIV of anterior instrumentation was T8 in 7 patients, T9 in 5, and T10 in 2. The LIV of anterior instrumentation was L1 in 9 patients and L2 in 5. No distal adding-on was observed.

### Radiographic findings

Radiographic coronal parameters at preoperative, postoperative 1 month, and final follow-up and the Δs are shown in Table 1. The anterior surgery corrected the preoperative main thoracic curve, thoracic apical translation, L4 tilt, and FDUV-CSVL at postoperative 1 month. The main thoracic Cobb angle increased a few degrees, but thoracic apical translation and C7-CSVL decreased at final follow-up. The median values of Δ L4 tilt, Δ disc angulation below LIV, and Δ FDUV-CSVL were almost zero, but the range varied from minus to plus (Δ L4 tilt: minus in 5 patients, Δ disc angulation below LIV: minus in 5 patients, ΔFDUV-CSVL: minus in 5 patients).

**Table 1:** Radiographic parameters

	Preoperative	Postoperative	Final follow-up	Δ
	Median (Range)	Median (Range)	Median (Range)	Median (Range)
Main thoracic Cobb angle (°)	55.1 (46.9—61.3)	18 (11.2—21.1)	19.5 (14.1—26)	2.4 (-2.8—6.2)
Thoracic flexibility (%)	69 (65.1—75.6)			
Thoracic apical translation (mm)	65 (59.1—73.9)	18 (8.8—22)	12 (6.3—19.5)	-1.5 (-5.3—0.9)
C7-CSVL (mm)	18 (3.2—25.2)	12 (0.5—24.5)	2.5 (-0.3—5.3)	-6.8 (-27.8—0.5)
L4 tilt (°)	16 (9.3—21.4)	2.2 (0—6.8)	3.3 (0—11.0)	0 (-1.4—1.9)
Disc angulation above UIV (°)	3.3 (2—7.1)	4.6 (3.8—5.5)	4.9 (3.5—6.2)	0.2 (-0.3—0.9)
Disc angulation below LIV (°)	3.5 (-3—4.7)	4.6 (2.3—6.5)	4.2 (2.5—7.7)	0.4 (-2.1—2.2)
FDUV-CSVL (mm)	21.4 (19.9—33.3)	5.15 (2.3—12.5)	5.8 (1.5—13.3)	0 (-2.1—4)
UIV-LIV Cobb angle (°)		-1.4 (-8.4—-0.3)	-0.2 (-3.1—1.5)	2.1 (0.5—4)

Range indicates interquartile range. Δ, the change from postoperative 1 month to final follow-up, *CSVL* central sacral vertical line, *UIV* upper instrumented vertebra, *LIV* lower instrumented vertebra, *FDUV* first distal uninstrumented vertebra

A negative number indicates either a tilt or translation or angle open to the left in the coronal plane

The median (interquartile range) UIV-LIV Cobb angle was -1.4° (-8.4—-0.3°) at postoperative 1 month and -0.2° (-3.1—1.5°) at final follow-up (minus sign denotes overcorrection). Anterior surgery in this study achieved overcorrection in 11 (79%) patients at postoperative 1 month.

The median screw angle (interquartile range) was 4.7° (-5.6—21°). The median screw angle in overcorrection group was 6.5˚ which was significantly larger than the angle in non-overcorrection group (i.e.—3.9˚, *p* = 0.029).

The median (interquartile range) value of TK, TLK and LL were 23.6˚ (11.9˚—34.8˚), 6˚ (2.5˚—14.8˚), and 61.5˚ (55.8˚—66.8˚) at preoperative, respectively. The angles at final follow-up were 23.8˚ (18.5˚—30˚), 4.9˚ (0.83˚—14.8˚) and 56.5˚ (50.8˚—62.9˚), respectively.

### Relationships between the UIV-LIV Cobb angle and the preoperative main curve-related parameters and screw angle

**Fig. 2:**
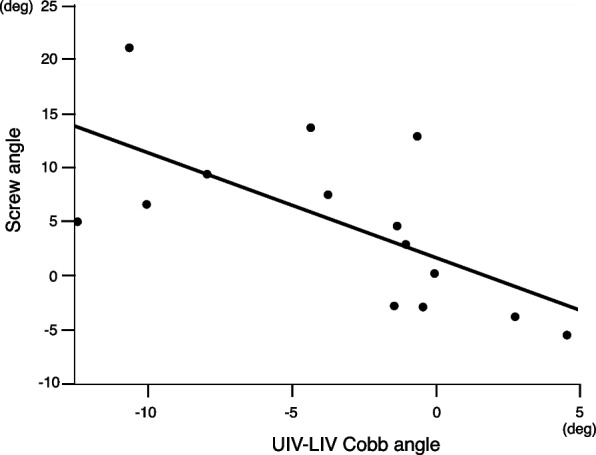
Relationship between the UIV-LIV Cobb angle and the screw angle. The UIV-LIV Cobb angle was significantly correlated with the screw angle. (*r*.^*2*^ = 0.42, *p* = 0.012)

The UIV-LIV Cobb angle was significantly correlated with only the screw angle on linear regression model analysis, leading to the following equation (Table 2, Fig. 2):

**Table 2:** Regression analyses of the UIV-LIV Cobb angle vs. preoperative main curve-related parameters and screw angle

	r^2^	p
Main thoracic Cobb angle (°)	0.04	0.51
Thoracic flexibility (%)	0.04	0.47
Thoracic apical translation (mm)	1.153e-5	0.99
Screw angle	0.42	0.012

*UIV* upper instrumented vertebra, *LIV* lower instrumented vertebra

$$\mathrm{Screw\ angle }= - 0.97 \times {\text{UIV-LIV}} \ {\text{Cobb angle }}+ 1.62\ ({r}^{2} = 0.42, p = 0.012)$$

### Relationships between the UIV-LIV Cobb angle and postoperative parameters

**Fig. 3:**
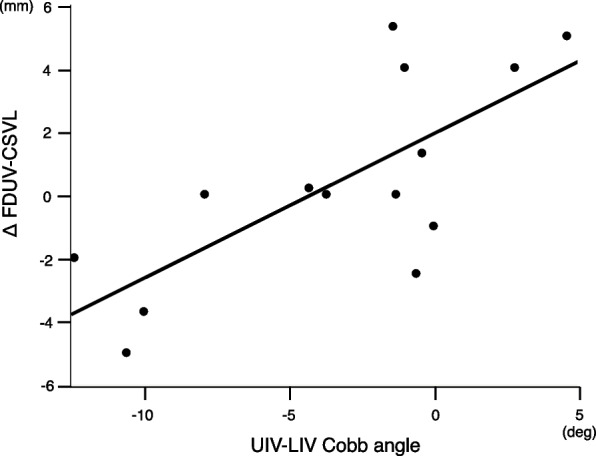
Relationship between the UIV-LIV Cobb angle and Δ FDUV-CSVL. The UIV-LIV Cobb angle was significantly correlated with Δ FDUV-CSVL. (*r*.^*2*^ = 0.53, *p* = 0.003)

There were no significant correlations with the UIV-LIV Cobb angle in the parameters of postoperative 1 month and final follow-up (Table 3). However, the UIV-LIV Cobb angle was significantly correlated with Δ FDUV-CSVL in a linear regression model, leading to the following equation (Table 4, Fig. 3):

**Table 3:** Regression analyses of the UIV-LIV Cobb angle vs. postoperative parameters

	Postoperative 1 month		Final follow-up	
	r^2^	p	r^2^	p
Main thoracic Cobb angle (°)	0.03	0.55	0.06	0.41
Thoracic apical translation (mm)	1.115e-5	0.99	0.03	0.54
Disc angulation above UIV (°)	0.06	0.41	0.2	0.11
Disc angulation below LIV (°)	0.28	0.054	0.06	0.42
FDUV-CSVL (mm)	0.04	0.51	0.04	0.51
L4 tilt (°)	0.25	0.07	0.11	0.26
C7-CSVL (mm)	0.005	0.81	6.036e-5	0.98

*UIV* upper instrumented vertebra, *LIV* lower instrumented vertebra, *FDUV* first distal uninstrumented vertebra, *CSVL* central sacral vertical line

**Table 4:** Regression analyses of the UIV-LIV Cobb angle vs. Δ

	r^2^	p
Δ Main thoracic Cobb angle (°)	0.03	0.54
Δ Thoracic apical translation (mm)	0.09	0.29
Δ Disc angulation above UIV (°)	0.1	0.27
Δ Disc angulation below LIV (°)	0.03	0.54
ΔFDUV-CSVL (mm)	0.53	0.003
Δ L4 tilt (°)	0.05	0.46
Δ C7-CSVL (mm)	0.006	0.79

*UIV* upper instrumented vertebra, *LIV* lower instrumented vertebra, Δ, the change from postoperative 1 month to final follow-up, *FDUV* first distal uninstrumented vertebra, *CSVL* central sacral vertical line

$$\Delta {\text{FDUV-CSVL}}= 0.46 \times {\text{UIV-LIV}}\ {\text{Cobb angle }}+ 1.91\ ({r}^{2} = 0.53, p = 0.003)$$

## Discussion

There are no past studies on the factors involved in achieving overcorrection in anterior fusion surgery for Lenke 1 AR type curves, and the details remain unclear. This is the first study to demonstrate that the screw angle (sum of screw insertion angles in UIV and LIV) is related to overcorrection. The effects of fusion with overcorrection on the postoperative course have also not been studied, but the present study showed for the first time the relationship between overcorrection and postoperative changes in FDUV-CSVL.

In the present study, to consider the factors involved in achieving overcorrection, the preoperative Cobb angle, flexibility, and apical translation were assumed to be factors in the main thoracic curve itself, and the screw angle was examined as a candidate factor related to the surgical technique. In idiopathic scoliosis, the preoperative Cobb angle and flexibility have been considered factors that affect correction of the deformity in surgery [7, 11], but in the present study, no correlation was seen with the UIV-LIV Cobb angle. The relative flexibility (flexibility: 69%) of the main thoracic curve that was examined in the present series, and the fact that cases of severe deformity with a large curve were not included (median Cobb angle: 55.1˚, interquartile range: 46.9˚-61.3˚), are thought to be involved as contributing factors. In the present series, the indication for anterior fusion was taken to be the apical vertebra at or below the T10/11 disc. Inami et al. examined the flexibility of the 1AR curve type and reported that the curve was more flexible in patients whose apical vertebra was at or below the T10/11 disc than in patients whose apical vertebra was T10 or higher [7]. Bernstein et al. stated that short anterior fusion with overcorrection for the lumbar curve improves trunk balance by rendering the vertebral bodies above and below the instrumented segment parallel to the sacrum [8]. We expected that the flexible thoracic curves being neighbor to the lumbar lesion could achieve this goal.

In the end, only the screw angle was correlated with the UIV-LIV Cobb angle (Fig. 2). Considering that a larger screw angle is thought to be a mechanism enabling overcorrection between UIV-LIV, the monoaxial screws were given greater angles than the endplate (i.e., the proximal screw was descending in the UIV, and the distal screw was ascending in the LIV) with sufficient release of the intervertebral space, and the rod and vertebral body were corrected to a rectangle or more by fixing the rod and screw in a rectangle (Fig. 1B, C). To create sufficient release between the vertebrae, sufficient removal of the intervertebral disc and cartilage endplate is important. Although the intervertebral disc and endplate were removed in this manner, TK and TLK did not deteriorate postoperatively.　Once a screw is inserted with an angle, it is important that it does not loosen during correction. For that purpose, it was decided to use a cantilever technique with a 4.5-mm titanium rod which has a little flexibility.

No parameters were significantly related to the UIV-LIV Cobb angle at postoperative 1 month and the final follow-up. However, a significant relationship was seen between the UIV-LIV Cobb angle and Δ FDUV-CSVL (Fig. 3). Thus, strongly overcorrecting at UIV-LIV was shown to produce a spontaneous correction with a balance in the lumbar coronal plane, which is a non-fused site below LIV, in the postoperative course (Fig. 1C, D). Spontaneous improvement in the global coronal balance (i.e. C7-CSVL) during the postoperative course following anterior fusion has been reported in the past study with Lenke 5 curve type. Yoshihara reviewed surgery outcomes of Lenke type 5, and in all 37 reports in that review, the coronal imbalance by C7-CSVL seen immediately after surgery was reported to have improved at the final follow-up [12]. In that review, it was reported that the disc angulation below the LIV increased immediately after the anterior fusion surgery, and it increased slightly even in the postoperative course. The disc angulation below the LIV and above UIV in the present study showed a change similar to that report (Fig. 1A, C, D). However, during the follow-up period of this study, the amount of change in angle was small and did not pose a major problem. On the other hand, with regard to the changes in FDUV-CSVL in the postoperative course, an increasing phenomenon has been reported in papers related to adding-on, but there are no past reports of a spontaneous decreasing phenomenon in the postoperative period. In the present study, cases in which FDUV-CSVL decreased in the postoperative period (i.e. from postoperative 1 month to final follow-up) were shown to exist (5 of 14 cases), and this is the first time that postoperative change of FDUV-CSVL showing a relationship with UIV-LIV Cobb angle has been reported.

The mechanism for spontaneous correction of FDUV-CSVL by overcorrection in the instrumented vertebrae must be considered. In general, for a decrease of FDUV-CSVL to occur, leveling of vertebral bodies caudal to LIV and a decrease in disc angle are thought to be necessary. Therefore, as a sub-analysis, a regression analysis of the relationship between Δ disc angulation below LIV or Δ L4 tilt and Δ FDUV-CSVL was performed, but no significant relationship was found. Thus, the mechanism for the spontaneous correction of FDUV-CSVL cannot be clearly shown here. The sum total of slight changes in the balance at multiple sites distal to the LIV, such as vertebral bodies caudal to LIV and disc angle, as well as the sacroiliac joint and lower limb compensation, may be expressed as spontaneous correction of FDUV-CSVL. In elucidating this mechanism, future studies with larger numbers of cases will be necessary.

This study has some limitations. First is the small number of patients. This was because the patients were limited to those with Lenke 1 AR curve type and the apical vertebra at or below the T10/11 disc. Second, this was a retrospective study, and we did not decide the degree of overcorrection for optimal curve correction preoperatively. In the future prospective study, it will be necessary to increase the number of patients and to validate the results obtained in this study. Third, there were no cases of severe AIS with a high cobb angle, so the effect of anterior surgery on such cases was unknown. Fourth, the relationships with quality of life (QOL) assessments and pain or other symptoms were not evaluated. Disc angulation below LIV and remaining FDUV-CSVL are reported to be involved in degeneration of discs at non-fused sites distal to LIV [13], and thus clinical evaluations during long-term follow-up will be needed in the future.

## Conclusions

In anterior fusion for patients with a Lenke type 1 AR curve type and apical vertebra at or below the T10/11 disc, overcorrection was achieved in the fusion range in 11 of 13 patients. The screw insertion angle was identified as a significant factor related to the achievement of overcorrection. In addition, overcorrection was shown to significantly affect changes over time FDUV-CSVL postoperatively. Overcorrection with anterior fusion is thought to be an effective method with which an improvement in the coronal balance over time can be expected.

## Data Availability

The data used and/or analyzed during this study are available from the corresponding author on reasonable request.

## Abbreviations

UIV: Upper instrumented vertebra
LIV: Lower instrumented vertebra
AIS: Adolescent idiopathic scoliosis
FDUV: First distal uninstrumented vertebra
CSVL: Central sacral vertical line
QOL: Quality of life
